# Relationship between protein-energy wasting in adults with chronic hemodialysis and the response to treatment with erythropoietin

**DOI:** 10.1186/s12882-019-1457-0

**Published:** 2019-08-14

**Authors:** Ailema González-Ortiz, Ricardo Correa-Rotter, Armando Vázquez-Rangel, Olynka Vega-Vega, Ángeles Espinosa-Cuevas

**Affiliations:** 1 Nephrology and Mineral Metabolism Department, Instituto Nacional de Ciencias Médicas y Nutrición Salvador Zubirán, Vasco de Quiroga No. 15, Col. Belisario Dominguez Sección XVI, Deleg. Tlalpan, 14080 Mexico City, CP Mexico; 2 School of Medicine, Universidad Nacional Autónoma de México, Ave. Universidad No. 3000, Col. Ciudad Universitaria, Deleg, Coyoacán, 04510 México City, CP Mexico; 3 Nephrology Department, Instituto Nacional de Cardiología Ignacio Chávez, Juan Badiano No. 1, Colonia Belisario Dominguez Sección XVI, Deleg. Tlalpan, 14080 Mexico City, CP Mexico; 4 Health Care Department, Universidad Autónoma Metropolitana-Xochimilco, Calz. Del Hueso No. 1100, Col. Villa Quietud, Deleg. Coyoacán, 04960 Mexico City, CP Mexico

**Keywords:** Protein energy wasting, Hemodialysis, Erythropoietin response

## Abstract

**Background:**

It is known that one of the leading causes of morbidity in chronic kidney disease (CKD) is the anemic syndrome. Although the pathogenic mechanisms of anemia are multiple, erythropoietin deficiency appears as the dominant factor. Patients in hemodialysis (HD) have a high prevalence of protein energy wasting (PEW) that may explains the poor response to Erythropoietin (EPO).

**Methods:**

Retrospective cohort study of patients on HD from January to December 2014. The participants were classified according to a diagnostic of PEW using the “Malnutrition Inflammation Score” (MIS) and bioimpedance analysis (BIA) measurement of body composition at the start of erythropoietin therapy and after 3 months of follow up. We performed descriptive statistics and analyzed the differences between groups with and without PEW considering their responsiveness. In addition, we calculated the relative risk of EPO resistance, considering *p* value < 0.05 as statistically significant.

**Results:**

Sixty-one patients ended the follow up. Both groups were similar in basal hemoglobin, hematocrit and other hematopoiesis markers (p = NS). Patients without PEW have a decrease risk for poor response to treatment with EPO (RR = 0.562 [95% CI, 0.329–0.961-]) than those with PEW. Finally, hemoglobin concentrations were evaluated at baseline and every four weeks until week 12, finding a statistically significant improvement only in patients without PEW according MIS (*p* < 0.05).

**Conclusions:**

PEW is an incremental predictor of poor responsiveness to EPO in HD patients, thus, it is important to consider correcting malnutrition or wasting for a favorable response to treatment with EPO.

## Background

One of the leading causes of morbidity in patients with advanced chronic kidney disease (CKD) is the presence of anemia. Although there are several pathogenic mechanisms for anemia in the CKD patient, Erythropoietin (EPO) deficiency appears as the dominant factor [1, 2]. There is a progressive decrease of the hematocrit (Hct); once the Glomerular Filtration Rate (GFR) falls below 60 ml/min /1.73m^2^; however, anemia is clinically assessable only after GFR has fallen to less than 30 ml/min/1.73m^2^ [3–5]. The addition of recombinant human erythropoietin (rHuEPO) to the treatment of anemia of renal origin has been the most important advance in this field. However, a percentage of patients responds poorly, or do not respond at all. The term “resistance” is meaningful here, since the serum concentrations of EPO required to obtain a response are extremely high, and the required dose of EPO is a predictor of mortality in this population [6, 7].

### Definition of poor response

In general, in most practice guidelines, the recommended range of Hb is 10-12 g/dl [5]. To reach this level, it is generally sufficient to administer initial doses of 50–150 Units/kilogram/week (U/kg/week) and then maintenance doses of 40 U/kg/week, as long as a proper iron store are assured in the patient [8, 9]. 

Patients of change in hemoglobin (Hb) level < 2% were operationally considered to have had a poor response, as compared with those in the upper change in hemoglobin level (2 to 15% or more). We assessed the mean hemoglobin level at 4 weeks (early phase), as well as the mean dose received after 12 weeks [10].

Since a 2% increase in the Hb concentration is likely to be within the variability range of Hb values in individual patients, this value is considered as no increase.The ESA responsiveness index (ERI) was defined as the weekly weight-adjusted EPO dose (U/kg/week) divided by the hemoglobin level (g/dL) and calculated monthly to investigate resistance to EPO treatment [11]. 

The most common causes of EPO resistance, among other, are secondary hyperparathyroidism, chronic inflammatory processes, and malnutrition. While iron deficiency is the leading cause of failure to respond to treatment with EPO; it must not be forgotten that active inflammatory or infectious processes can temporarily block the response to iron therapy and, thereby, to EPO [12–14]. It is important to remember that the presence of anemia in the HD population, is considered one of the main causes of morbidity, closely related to a decrease in appetite and to a hypercatabolic state, all of which may induce a state of malnutrition and inflammation also known as protein-energy wasting (PEW) [15–17].

Recent literature suggests that PEW is an independent factor for EPO responsiveness when initiating treatment with erythropoiesis stimulating agents (ESA) [18–20]. With the presence of PEW, there is a less response to EPO therapy; however, results are not yet conclusive.

## Methods

We conducted a retrospective cohort study including adult patients on HD from our Institute from January to December; ages > 18 years, with Kt/V > 1.2 and/or URR > 65%, serum ferritin > 100 ng/dl and transferrin saturation > 20%, and an individualized prescription of EPO. Patients were excluded if they had an immunologically mediated disease as lupus erythematosus and were in treatment with remission induction therapy (patients who after 3 months might recover renal function), if they had any metallic prosthesis that could interfere with bioelectrical impedance, if they had an active infectious disease, or if they had been in an emergency room or hospitalized at any point during the previous 3 months. Clinical, biochemical and treatment data, including EPO dose was obtained through medical records that are done monthly. Malnutrition Inflammation Score (MIS) and bio impedance vector analysis (BIVA) [22] are done routinely every 3 months in this population. All participants were followed up for 12 weeks. PEW was defined as a MIS [15] of > 7 [18, 21] or phase angle ≤5°.

Patients with an increase in hemoglobin level ≤ 2% is considered a non-significant change or poor response after the EPO treatment.

The nutritional status and body composition of the participants were analyzed by BIVA, using conventional measurements of bio impedance analysis (BIA); resistance (R), reactance (Xc) and Phase Angle; the data were then plotted using the method of bioelectrical impedance vectors normalized in Z-score, that is, Z (R) and Z (Xc) [22–24].

The protocol was approved by the Human Research and Ethics Committee of the Instituto Nacional de Ciencias Médicas y Nutrición Salvador Zubirán; (reference 1407). Data and material availability of the study will be provided if requested.

### Statistical analysis

Data were summarized using proportions; means [±standard deviation (SD)] or medians (inter-quartile range) as appropriate using the Shapiro-Wilk for testing normality. To find differences between patients with or without PEW, we used *Xi*^2^, Mann-Whitney’s U test or Student’s t, according to their distribution. The correlation between phase angle by BIA or MIS and hemoglobin concentration was evaluated using Pearson’s or Spearman’s correlation coefficients. A test of repeated measures ANOVA or Kruskal Wallis was performed to compare changes in hemoglobin levels during the period of the study. We estimated the relative risk for poor response to treatment according to PEW diagnosis. To compare the confidence ellipses of the impedance vectors we used Hotelling’s T^2^ test. A *p* < 0.05 was considered as statistically significant. The data were analyzed using SPSS 20.0 (SPSS Inc., Chicago, IL, USA).

## Results

**Fig. 1:**
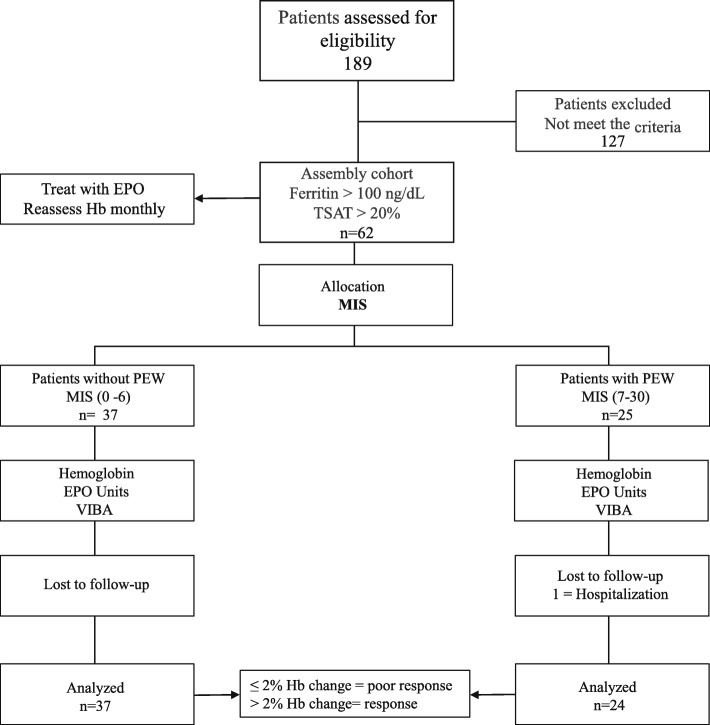
Flowchart showing the participation process

The medical records of 189 patients from our HD Clinic were evaluated retrospectively (Fig. 1). One hundred twenty-seven patients did not meet the inclusion criteria.

A total of 61 patients were included in the study, of which 37 had not PEW according MIS. General characteristics of the population are shown in Table 1. As expected, per definition of the two groups, statistically significant differences were found between the two groups in variables associated with nutritional status such as weight, body mass index (BMI), serum creatinine, serum albumin and potassium also we found that the patients with PEW had a higher ERI (IU/kg/week) (*p* = 0.04). Patients with PEW according to MIS had lower concentrations of serum creatinine than those without PEW. No significant differences in Hb, Hct, serum iron, transferrin saturation (TSAT), serum ferritin (Table 1). Patients that had low iron levels not necessarily were given iv iron, but we registered a 57.5% of patients that did received IV iron and the dose was at 100 mg weekly X 10 doses.

**Table 1:** General characteristics of the population. Anthropometric and demographic characteristics

VARIABLE (%)	without PEW *n* = 37 (60.6)	PEW *n* = 24 (39.4)	p value
Age (year)	30 (20–77)	28.5 (19–63)	0.172
Time on dialysis (months)	8 (3–50)	4.5 (3–32)	0.044
Weight (kg)	57.5 (43–105)	48 (35–87.7)	0.015
Height (meters)	1.60 ± 0.08	1.57 ± 0.09	0.259
BMI (kg/m^2^)	23.27 ± 4.9	20.35 ± 3.3	0.015
KT/V	1.67 ± 0.34	1.77 ± 0.40	0.311
URR %	79.73 ± 6.02	80.24 ± 7.9	0.800
Creatinine (mg/dl)	10.8 (4.19–20.5)	6 (2.52–15.84)	0.001
Sodium (mmol/L)	138.19 ± 3.55	138.05 ± 3.46	0.324
Potassium (mmol/L)	5.128 ± 0.59	4.50 ± 0.89	0.009
Phosphorus (μg/L)	4.98 ± 1.76	4.27 ± 1.47	0.098
Serum albumin (g/dl)	3.46 ± 0.521	3.10 ± 0.47	0.008
Glucose (mg/dl)	112.22 (72–194)	105 (72–597)	0.696
Uric acid (mg/dl)	7.48 ± 1.51	7.36 ± 2.35	0.787
Hemoglobin (g/dl)	8.9 ± 1.78	9.34 ± 1.18	0.279
Hematocrit(%)	27.26 ± 5.5	28.37 ± 3.93	0.237
PTH (pg/mL)	498.4 (26–1825)	226 (25.8–1005)	0.046
Serum Iron μg/dL	75 (42–195– 104)	76 (27–202)	0.922
Transferrin saturation %	35.4 (20–61)	32 (20–96.5)	0.842
Serum ferritin	315.7 (100–1270)	461.8 (118.8–1296.7)	0.142
Epo U/kg/week	112 (32.5–276.5)	136 (66.5–270)	0.040
Impedance characteristics			
MEN	*n* = 11	*n* = 9	
Resistance R (Ohms)	522.6 ± 102.3	588 ± 115.4	0.203
Reactance Xc (Ohms)	52.9 (28.4–81)	50.5 (24.9–99.3)	0.456
Phase Angle (°)	5.95 ± 1.27	4.88 ± 1.28	0.083
Resistance/height(Ohms/meters)	312.08 ± 64.15	457.7 ± 79	0.185
Reactance/height (Ohms/meters)	31.1 (16–48.2)	29.7 (14.3–64.9)	0.656
WOMEN	*n* = 26	*n* = 15	
Resistance R (Ohms)	638.15 ± 112	724.2 ± 154.5	0.072
Reactance Xc (Ohms)	62.9 (21.3–104)	49 (23–68)	0.076
Phase Angle (°)	5.2 ± 1.69	4.05 ± 1.33	0.020
Resistance/height(Ohms/meters)	406.7 ± 76.5	474.4 ± 106.1	0.041
Reactance/height (Ohms/meters)	40.7 (12.45–67.9)	31.2 (15.7–44.9)	0.127

Continuous variables are expressed as mean ± standard deviation, non-parametric as medians or inter-quartile ranges The categorical variables were compared using χ2 test and continuous variables were compared using the T or U Mann-Whitney test according to the variable

The BIA analysis of the studied population with and without PEW and subdivided by gender are shown in Table 1. Phase angle was higher in patients without PEW even though statistically significant differences were found only in women. In both genders, resistance is slightly lower in patients without PEW, indicating more lean body mass with statistically significant difference only in women (Table 1).

**Fig. 2:**
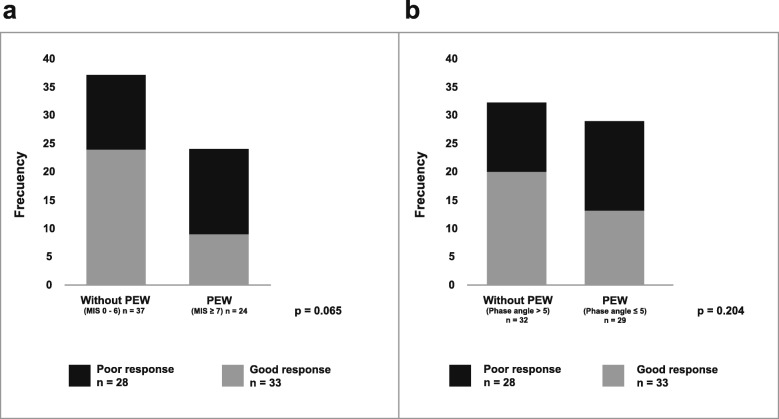
Relationship between the diagnosis PEW by MIS ≥7 (**a**) and Phase Angle ≤5 (**b**) with the response to treatment with EPO. (Xi^2^ test was performed to determine differences between groups)

When analyzing the relationship between PEW by MIS score and the response to treatment with EPO (Fig. 2a), we found that 33 out of 61 patients responded satisfactorily, being the majority 72.7from the group of patients without PEW. In patients with PEW as defined by MIS, 53.6% had a poor response to EPO vs. only 46.4% of those patients without PEW showed hyporesponsivenes to EPO (*p* = 0.065). A non-significant change in the same direction was observed in patients with PEW defined by phase angle (33.3 vs 66.6%, *p* = 0.204) (Fig. 2b).

After analyzing the characteristics of the patients according to the response to treatment with EPO, it was found that those patients with good response were those with the best nutritional status measured by MIS (*p* < 0.05), the median change of patients with good response it was 17.9% vs − 7.7% in those patients with a poor response (*p* < 0.001) Table 2.

**Table 2:** Characteristics of the population according EPO response

VARIABLE (%)	Response *n* = 33			Poor response *n* = 28			p value Final vs Final
	Basal	Final	∆	Basal	Final	∆	
BMI (kg/m^2^)	21.96 ± 3.9	21.7 ± 3.66	0.22	22 ± 5.18	21.2 ± 3.9	− 0.14	0.674
MIS score	5 (1–14)	4 (0–10)*	− 1.57	7 (1–13)	6 (1–11)	0	0.002
Phase Angle (°)	5.5 (2.1–7.6)	5.5 (2.5–13)*	0.74	4.7 (2.3–8.5)	4.8 (2.2–8.1)	−0.26	0.1
Hemoglobin (g/dl)	8.8 ± 1.5	10.6 ± 1.7 *	Change% 17.9 (3.2–75.4)	9.5 ± 1.6	8.5 ± 1.5 *	Change% − 7.7 (− 39.1–1.6)	0.001

Continuous variables are expressed as mean ± standard deviation, non-parametric as medians or inter-quartile ranges

* *p* < 0.05 Basal vs Final

When estimating the relative risk (RR) for EPO response, we found that those patients without PEW by MIS (≤6) and by phase angle, (≥5), have a risk of response to treatment with EPO, (RR = 0.562 [CI 95% 0.329–0.961]) and (RR = 0.068 [CI95% 0.39–1.184]) as compared to those with PEW; however, the phase angle RR did not reach statistical significance.

**Fig. 3:**
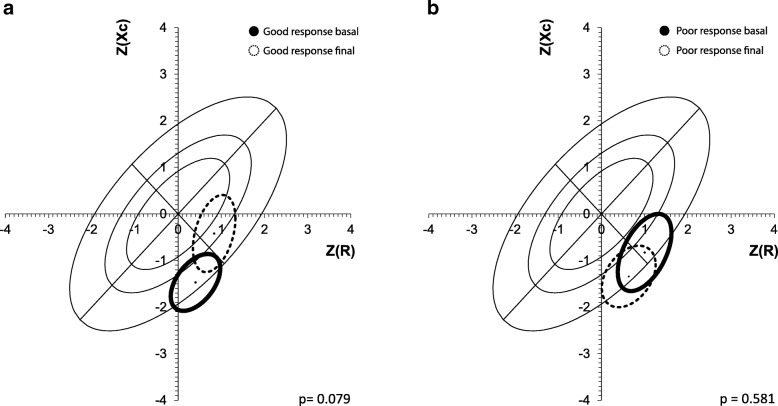
Relationship between BIVA and treatment with EPO according to the good (**a**) and poor (**b**) response

With respect to the impedance vector analysis, we found that patients with poor response to EPO, showed a shift on the low–right side of the short axis of the vector outside the 75th percentile of the tolerance ellipses, indicating a certain trend to cachexia, although we did not find a statistically significant difference (Hottelling T^2^ Test; *p* < 0.58), (Fig. 3b). Patients with good response to EPO improved their body composition, migrating towards the center of tolerance ellipses indicating better dry weight and body mass although we did not find a statistically significant difference (*p* = 0.079), (Fig. 3a).

**Fig. 4:**
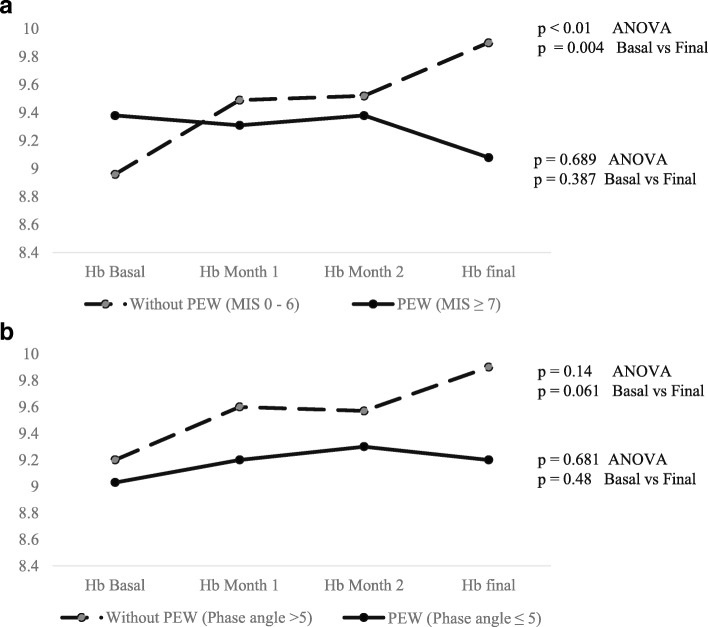
Longitudinal comparisons of Hb concentrations during the study period according MIS (**a**) and Phase Angle (**b**)

Finally, hemoglobin levels were evaluated at baseline and every four weeks until week 12, finding a statistically significant improvement only in patients without PEW diagnosed by MIS (Fig. 4a) (*p* < 0.01) and phase angle (Fig. 4b).

## Discussion

Treatment of anemia of patients with CKD has significantly changed over 20 years ago with the addition of recombinant human erythropoietin. Over 90% of patients that receive EPO respond to treatment; however, many factors may block or diminish the response to this hormone replacement. Once iron deficiency and active bleeding are excluded, the diagnosis of EPO resistance is established, and a multiplicity of factors could be responsible for this [25].

The most commonly reported causes of lack of response to EPO treatment are iron deficiency, secondary hyperparathyroidism, chronic inflammatory processes, and malnutrition. Iron deficiency appears as the most frequent cause of failure to respond to treatment with EPO; however, it must not be forgotten that active inflammatory or infectious processes can temporarily block the response to iron therapy and therefore, to EPO [12–14]. That is why an evaluation of PEW is always relevant in this population. The concept of PEW, proposed by the International Society of Renal Nutrition and Metabolism, refers to a condition that encompasses the multiple nutritional and catabolic alterations that occur in chronic kidney disease and that are associated with morbidity and mortality. Although insufficient food intake due to lack of appetite and dietary restrictions contribute to PEW, other factors contribute to the development of this syndrome, such as alterations induced by uremia, increases in energy expenditure, persistent inflammation, acidosis, and multiple endocrine disorders that cause a state of hyper-metabolism, leading to excessive catabolism of muscle and fat [16].

Several studies have assessed the response to EPO according to various factors; however, very few studies have had as main objective to assess the nutritional status of the subjects, measuring it instead through isolated variables. Among the studies that assess nutritional status through isolated variables is one published by Kudoh et al. who assessed the effect of carnitine as therapy for overcoming the resistance to EPO; they found that less than 50% of studied patients responded to treatment with EPO plus carnitine, showing a serum albumin concentration of 3.7 ± 0.2 vs 3.6 ± 0.02 g/dL in poor responders, although these differences were not statistically significant [26]. However, the present study do not found a differences statistically significant according albumin and EPO response (3.2 ± 0.41 vs 3.4 ± 0.63 *p* = 259). A cross-sectional study by Locatelli et al. that aimed to assess the response to treatment with EPO, found that patients with a lower Index of Response to Erythropoietin (IRE) had a better nutritional status as assessed by BMI (24.9 ± 4.3 vs 21.3 ± 3.8 m/kg2, *p* < 0.001) and albumin (4.2 ± 0.4 vs 4.1 ± 0.5 g/dl p < 0.001) [7], contrary to what we observed in this study, in which patients who did respond to treatment had a similar BMI than those who did not respond satisfactorily to treatment (21.96 ± 3.9 vs 22 ± 5.18 *p* = 0.645)). Nevertheless, it is worth noting that Locatelli et al. ranked their results according to the IRE, whereas in this study the outcome variable was the response, or lack of response, to treatment according to the diagnosis of PEW.

Only a few studies have had the main purpose of assessing the relationship between PEW and the response to treatment with EPO, such as that conducted by Kalantar et al., in which the main goal was to find the association between serum ferritin and markers of inflammation, nutrition, and iron in hemodialysis. They found that patients with lower Hb levels (10.9 ± 1.7 vs 10.3 ± 1.18 g/dL *p* < 0.4) were also the ones with worse nutritional status, but the differences were not significant. They also included the MIS score as a variable, finding that patients with malnutrition by Subjective global assessment (SGA) according to were also those with the highest MIS score (SGA 1 = MIS 4.0 ± 1.2, SGA 2 = MIS 7.7 ± 1.8 and SGA 3 = 13.4 ± 3.0) [27]. This study served as the basis for establishing the cutoff point in MIS with respect to the diagnosis of PEW (MIS 0–6 = no PEW vs MIS ≥7 = PEW) in this research. In another study, the same group of researchers led by Kalantar et al. evaluated the effect of PEW on the poor response to treatment with EPO in patients undergoing HD. They suggested that the presence of low concentrations of cholesterol, prealbumin, and iron, among others, has a major effect on the poor response to treatment (measured by IRE), since they found that those with a lower IRE had a lower MIS score, while a higher IRE corresponded to higher total scores (5.8 ± 3.4 vs 7.5 ± 4.0 *p* < 0.001). Regarding the BMI, they reported that patients with lower IRE had a better BMI compared to those with a higher IRE, who had a lower BMI (27.7 ± 7.6 vs 25.3 ± 5.5) [19].

Recently, a study of 754 HD patients by Rattanasompattikul et al., with complete nutritional and inflammatory markers data, considered wasting as an independent factor for resistance to EPO. They found that elevated levels of inflammatory markers and low levels of nutritional markers were independent predictors of a decreased response to EPO [18]. MIS scores lower than 6.4 were associated with a poor response to treatment with EPO (p < 0.001). We describe similar results in our study, where the cutoff for diagnosis of PEW was MIS ≥ 7.

The pro-inflammatory cytokines are potent inhibitors of erythropoiesis in vivo and in vitro [4]. The inhibition of erythropoiesis by cytokines such as TNF-α and IFN-γ is also important in the development of resistance to EPO, as well as to Interleukin-6 (IL-6) and C-reactive protein (CRP). These cytokines participate in the genesis of anemia through different mechanisms: accelerating the apoptosis of erythroid precursors, decreasing the number of EPO receptors in them, producing a relative decrease in the synthesis of EPO by the kidneys, increasing the synthesis of hepcidin and inducing erythrophagocytosis [26, 28–30]. One limitation of our study is that we did not have any data about CRP or other inflammation markers, as inflammation is a clear confounder as it often associated with both EPO poor response and PEW.

Since these cytokines have a similar sensitivity for the diagnosis of inflammation as MIS, we decided to make an independent evaluation of the predictions of MIS. Patients diagnosed without PEW (0–6 points of the sum of items MIS) have a 95% decrease risk of poor response to treatment with EPO than those with PEW (RR = 0.068 [CI95% 0.39–1.184]).

Another way to evaluate PEW and cachexia (severe form of PEW) is based on bioelectrical impedance vectors, or the components of the body’s electrical properties (resistance, reactance, and phase angle) [22, 23, 31–34]. Colin et al. found that patients with a phase angle of 4.65 ± 1.23° had a Hb of 12.33 g/dl ± 0.67 g/dl and an Hct of 37.28% ± 2.49 g/dl, while those patients with a phase angle of 5.48 ± 1.17° had an Hb concentration of 15.09 ± 1.55 g/dl and an Hct concentration of 45.01 ± 4.70%; these differences were statistically significant for each indicator (*p* < 0.001). In addition, the final measurement showed that those patients with a phase angle greater than 5 responded satisfactorily to treatment with EPO, while those with a lower phase angle did not respond to the treatment. Patients with resistance to EPO had a vector pattern indicating a trend to cachexia (p < NS), while patients who responded to treatment had a normal vector pattern. The major limitation of our study is that it was a retrospective, single center study, and the relatively small sample size.

## Conclusions

The results of this study suggest that PEW is an incremental predictor of poor responsiveness to EPO in HD patients. It is important to consider that all patients should have a careful nutritional and body composition assessment, to implement an exhaustive intervention if required and thus favor an adequate response to the EPO and consequently reduce the presence of anemia. However, it is necessary to do more studies with larger sample size to be able to verify this hypothesis, and control with measurement of inflammation.

## Data Availability

All the authors agree to provide the analysis database as well as any material in case the editorial committee requires it.

## Abbreviations

BIA: Bioimpedance analysis
BIVA: Bioimpedance vector analysis
BMI: Body mass index
CKD: Chronic kidney disease
CRP: C-reactive protein
EPO: Erythropoietin
ERI: ESA responsiveness index
ESA: Erythropoiesis stimulating agents
GFR: Glomerular Filtration Rate
Hb: Hemoglobin
Hct: Hematocrit
HD: Hemodialysis
IFN-γ: Interferon-γ
IL-1: Interleukin-1
IL-6: Interleukin-6
IRE: Index of Response to Erythropoietin
MIS: Malnutrition Inflammation Score
PEW: Protein-energy wasting
R: Resistance
rHuEPO: Recombinant human erythropoietin
SGA: Subjective global assessment
TNF –β: Tumor necrosis factor-β
TNF-α: Tumor necrosis factor alpha
TSAT: Transferrin saturarion
U/kg/week: Units/kilogram/week
Xc: Reactance
